# From egalitarianism to veiled selectivity: education, social mobility and the reproduction of inequalities in contemporary Argentina

**DOI:** 10.3389/fsoc.2026.1758238

**Published:** 2026-02-11

**Authors:** Sandra Ziegler, Manuel Giovine

**Affiliations:** 1 Area Educación, Facultad Latinoamericana de Ciencias Sociales (FLACSO), Buenos Aires, Argentina; 2 Departamento de Sociología y Antropología, Universidad de La Laguna, San Cristóbal de La Laguna, Spain

**Keywords:** egalitarianism, private education, reproduction of inequalities, school segregation, social mobility, veiled selectivity

## Abstract

This article examines how the Argentine education system, historically characterised by an egalitarian matrix and an open access model, contributes to processes of stratification and social mobility through covert social selection mechanisms. To this end, historical statistical data showing the evolution of educational distribution by socioeconomic level are presented, and performance patterns in language and mathematics are analysed according to management sector and income quintile. This evidence highlights the persistence of inequalities in secondary school completion and academic performance, and shows that the non-selective nature of the system coexists with forms of stratification that are expressed through differentiated school trajectories. The article also includes the results of recent research based on a survey of 588 families in different cities across the country, which provides insight into the strategies deployed through school choice to mitigate downward social mobility. The analysis shows that families’ choice of private schools, particularly at the secondary level and especially among the lower classes and impoverished middle classes, is a response to the need to ensure continuous and successful school trajectories in a context where selectivity operates through academic performance. The identification of two large family groups within the private demand, with different positions in the social space, reveals that private education fulfils various functions: as a mechanism of social closure for consolidated sectors and as a preventive strategy for groups in transition seeking to ensure educational stability and better academic results. In summary, the paper provides a structural reading of recent transformations in the relationship between education and inequality in Argentina. Although the open access model that historically distinguished the system retains its normative and symbolic validity, its current functioning is strained by mechanisms of veiled selection that are linked to social stratification. By showing how the choice of private secondary schools has become a strategy for coping with uncertainty and avoiding interrupted school trajectories, the article highlights a process of increasing segmentation that is reshaping the educational landscape and contributing to the redefinition of contemporary forms of social reproduction and differentiation through the role played by schooling processes.

## 1 Introduction

For much of the 20th century, Argentina was characterised as a relatively egalitarian society open to social mobility. This image was based on structural historical processes: the upward mobility of the first generations of European migrants, industrial development in the mid-20th century, the expansion of formal wage employment, and the consolidation of social rights during the Peronist governments of the 1940s and 1950s (Kessler and Assusa, 2025). Free education, the strengthening of trade unions, and expanded access to public goods and services cemented a model of integration that was interpreted as a sign of an expanding middle class society (Germani, 1963; Torrado, 1992).

This “egalitarian impulse” (Torre, 2024) marked the Argentine social structure with a class matrix of upward mobility, and this dynamic defined a profound difference with the hierarchical and rigid social structure model of the vast majority of Latin American countries, including its closest neighbours (Brazil, Bolivia, Chile, and Paraguay). In Argentina, the urban working classes, supported by factory employment and trade union organisation, constituted a dynamic and visible core, while the middle classes grew at the pace of state development and import substitution industry (Dalle, 2016). The configuration of this structure explained Argentina’s exceptional position in the region (Benza, 2016), where inequality appeared to be less pronounced.

The education system in Argentina was a central driver that accompanied the development of this social structure, and indeed, classic sociological studies such as that of Germani (1963) point out that, during the first seven decades of the 20th century, there was a permanent dynamic of intergenerational social mobility that was accompanied by an increase in the educational credentials of the population. Hence, the increase in years of schooling was interpreted as a fundamental strategy for upward social mobility. This framework was linked to a system of compulsory, mass, free primary education and a secondary school system which, although selective in its early days (Tedesco, 1986), gradually expanded access to broader social sectors (Almeida et al., 2017).

The egalitarian matrix and the belief in social advancement through education in the country began to erode with the economic, political and social changes that began in the mid-1970s. The military dictatorship that began in 1976 implemented a programme of deindustrialisation, economic liberalisation and repression that profoundly affected living conditions. The democracy restored in 1983 inherited a much more fragmented and impoverished society. It was then that sociological studies of the time proposed the notion of the ‘new poor’, made up of impoverished middle classes, distinct from traditional structural poverty (Murmis and Feldman, 1992; Minujin, 1995).

During the 1990s, the neoliberal programme deepened inequality with policies of privatisation, deregulation and labour flexibility. Although there was a modernisation of the occupational structure and an increase in skilled jobs, once again the effects did not have the expected impact on living standards and job stability; the new jobs were often poorly paid or unstable, generating what Kessler and Espinoza (2003) called “spurious mobility,” occupational promotions without economic improvements or even with deteriorating living conditions.

This phenomenon revealed a fracture in the traditional mechanisms of social integration, where education no longer guaranteed quality, well-paid jobs, and formal work did not ensure well-being. At the same time, a hard core of structural poverty emerged, accompanied by new forms of social exclusion (Svampa, 2005; Auyero, 2001).

With the post-neoliberal cycle that began in 2003, the inclusion policies implemented sought to reverse some of the trends of the 1990s and, to a certain extent, broadened the expectation of social mobility through education, which was still in force. The long historical journey of Argentina’s social structure reveals a persistent tension between a deeply rooted egalitarian imaginary (Terán, 2002; O’Donnell, 1976) and the structural transformations that, with ups and downs, have eroded its material foundations. From upward mobility as a trend to spurious mobility as a paradox, inequality in Argentina challenges not only its institutions but also the very foundations of its social identity.

In recent decades, we have witnessed an unprecedented process of massification at the secondary and higher education levels (university and non-university) that has had multiple effects on the social reality of individuals. Families from working-class sectors are accessing secondary school for the first time, and middle-class families with low educational qualifications are seeing their sons and daughters reach university for the first time. The structural effects are significant: serious difficulties in completing studies at both levels, a trend towards the privatisation of education as an *ad hoc* strategy to sustain the ideal of social advancement through schooling, and upper classes accumulating postgraduate credentials and generating new, increasingly restrictive exclusive circuits (Giovine, 2022).

This article proposes to account for the dynamics of the Argentine education system and its association with egalitarian matrices, demonstrating in its evolution the configuration of a structure that channels expectations of social mobility through an open model, insofar as there are no selective entrance exams throughout all levels of the education system (including access to university) (Tiramonti and Ziegler, 2008). Unlike other countries in the region, where tests are administered at the end of secondary education (such as in Chile or the Vestibular in Brazil), in Argentina, free enrolment in higher education levels is permitted, without any restrictions. This dynamic, associated with egalitarian expectations, effectively allows certain sectors of the population to access levels that were forbidden to previous generations. However, for the vast majority, a form of veiled selection operates: a social selection mediated by the education system, which takes a “soft” form (Di Piero, 2022), in that it occurs throughout the school career through the dropout of those who fail to meet academic performance requirements.

This paper seeks to provide a structural reading of recent transformations in the relationship between education and inequality in Argentina. Although the open access model that historically distinguished the system retains its normative and symbolic validity, its current functioning is strained by selective mechanisms that are linked to social stratification. By showing how the choice of private secondary schools has become a strategy for some families to cope with uncertainty and avoid interrupted school trajectories, the article highlights a process of increasing segmentation that is reconfiguring the educational landscape and contributing to the redefinition of contemporary forms of social reproduction and differentiation in the country.

In short, this work seeks to provide a historical and structural perspective on the relationship between education and inequality in Argentina, framing the specificity of the educational system as part of an egalitarian matrix that, although weakened, continues to shape social expectations around social mobility.

To this end, it is worth revisiting Sandel’s (2020) argument, who astutely demonstrates how, in the case of the United States, meritocracy, historically presented as the main mechanism for social mobility and as an alternative to inheritance or a destiny determined by class, acquired a legitimising character for the social order. In the case of Argentina, this meritocratic ideal found equalising support in formal education and state institutions for much of the 20th century. However, as schooling expanded and became more democratic at all levels, this levelling role seems to have shifted partially to the private sector. In this scenario, working-class and middle-class sectors make significant economic efforts to access these institutions in order to sustain expectations of social mobility. In this article, we explore this hypothesis.

The paper is structured in the following sections: we present the methodology used to obtain the data that gave rise to this article. Subsequently, we analyse official statistics to characterise the distribution of the population across different educational levels and the demand for private education at the secondary level. Next, we review the results of recent empirical research which, based on a survey of families, reveals school choice strategies and internal divisions within the private sector, identifying types of families and their motivations for choosing private education. The survey findings complement the statistical trends presented. Finally, the concluding discussion integrates the findings to address the role of private education in validating the dynamics of the social structure, concluding on the contribution of the choice of private education in reinforcing school segregation. In closing, future lines of research arising from the advances of this study are proposed.

## 2 Methodology

To account for the dynamics of the Argentine education system and its association with egalitarian matrices, and to discuss the role of education in validating the dynamics of the social structure, the research draws on two large bodies of information: official statistical data (both historical and recent) and the results of recent empirical research.

To analyse the demand for private education in large urban agglomerations in Argentina since the beginning of the 21st century, data from the Permanent Household Survey (Encuesta Permanente de Hogares-EPH) conducted by the National Institute of Statistics and Censuses (Instituto Nacional de Estadísticas y Censos-INDEC), the country’s official public statistics agency, were used. The EPH is the main continuous statistical operation in Argentina. It is a sample survey of urban households that collects information on employment, income, education, housing, and demographic information. Its coverage includes the country’s main urban agglomerations, which account for approximately two-thirds of Argentina’s population. Its data are used to produce the most commonly used labour indicators, such as activity, employment, and unemployment rates, as well as statistics on income distribution and the social characteristics of households. The EPH uses a stratified probability design with panel rotation, which allows for the combination of cross-sectional and short-term longitudinal information. As it is the official source for measuring Argentina’s socioeconomic situation, its results constitute the empirical basis for academic studies, public policy analysis and international comparisons.

Data from “Operativo Aprender,” a national educational assessment system implemented since 2016, was also used to analyse performance in language and mathematics in the third and sixth grades of primary school and the third and sixth years of secondary school, adding social sciences and natural sciences, sometimes using samples and sometimes using a census. The data allow for analysis of state and private education and according to the socioeconomic level of the population (among other variables). This operation assesses learning and surveys socio-educational conditions through questionnaires to students, teachers, and principals. In some cases, it is sample-based and in others, census-based. The data included in this study correspond to the year 2024, when the operation was census-based and involved 379.050 sixth-grade students from 11.846 schools.

The analysis of learning outcomes presented in the following sections should be interpreted with caution. In particular, comparisons between students from the lowest income quintile attending private and state schools do not involve strictly equivalent populations, as relevant unobserved factors (such as cultural capital, prior educational trajectories, or family expectations) may influence school choice and academic performance. Therefore, the associations identified should not be read as direct causal effects of school management, but rather as empirical regularities emerging from specific analytical decisions and available variables. Alternative methodological choices or additional controls could lead to different interpretations.

This paper also draws on the results of recent research that reports on the educational strategies employed by families when selecting a school for their children in order to mitigate the risks associated with social decline.

For this study, a quantitative survey was conducted using an anonymous, self-administered online questionnaire aimed at parents of students enrolled in the third year of secondary school in 2022 in private institutions in the Autonomous City of Buenos Aires (CABA) and the provinces of Buenos Aires, Córdoba, Santa Fe, and Tucumán. Intentional multistage sampling was used: first, provinces with a high relative participation of the private sector in secondary education were selected, using as a proxy the number of sections per school published on the poblaciones.org platform, based on data from the Ministry of Education. In each province, cities of different sizes were chosen to capture heterogeneities associated with urban scale and the diversity of private education offerings. The survey was administered through 32 schools (9 in Buenos Aires, 6 in CABA, 6 in Córdoba, 6 in Santa Fe, and 5 in Tucumán), which sent the form to the families of third-year students; 588 responses were obtained. The sample is non-probabilistic and does not claim to be representative of all families with children enrolled in private schools, but rather to offer empirical evidence on their school choice strategies (Ziegler and Giovine, 2025).

The questionnaire combined closed and open-ended questions and was administered using Google Forms, which allowed for the definition of conditional response paths and minimised navigation errors. Special care was taken with the length (no more than ten minutes to complete) and the clarity of the questions, given that no interviewers were involved. The sample included private religious and non-religious institutions, with full or partial state subsidies and without subsidies, in order to capture families from different socioeconomic levels. For processing and analysis, interactive data dashboards were developed that integrate information from sections, responses, and metadata, facilitating dynamic data exploration, hypothesis validation, and analysis.

The data from the questionnaires administered to families were processed using Multiple Correspondence Analysis (MCA), as applied by Bourdieu in *Distinction* (Bourdieu, 1988). MCA is a statistical technique that allows the relationship between categorical variables to be analysed and visualised in a multidimensional space, revealing the underlying structure and regularities in the data. Its use allows the social and symbolic dynamics that structure social space to be mapped and understood.

This method was chosen because it allows for the simultaneous analysis of a large number of categorical variables and is particularly powerful for studies with complex and multidimensional data. It allows for the clear visualisation of relationships between variables, as the multidimensional space generated by MCA facilitates the interpretation of results. The identification of groups of cases with similar characteristics facilitates the understanding of their internal qualities and, in turn, highlights the differences between them. In this way, the technique contributes to the construction of typologies and classifications based on the available empirical data.

The potential of ACM lies in its ability to identify patterns of association between variables, examining significant relationships and oppositions; to construct a multidimensional space where the proximity between points (cases and categories) reflects the similarity in their characteristics; to group cases with similar characteristics into *clusters* and to develop hierarchical classifications to construct typologies.

The application of ACM followed three stages. An objectivist stage that allows the construction of the space of families’ positions based on structural variables. A second subjectivist stage focused on analysing the positions taken by these families according to their perceptions and assessments. A final relational stage aimed at relating perceptions and assessments to position in social space.

The variables used in shaping the space included: the highest level of education attained by the parents, the type of school attended by the responsible adult, the number of people and rooms in the home, the availability of computer resources and connectivity (computers, mobile phones, internet), the percentage of monthly income allocated to school fees, the subsidy received by the institution, the religious nature of the institution, and the size of the city.

Using this technique, a hierarchical and ascending classification scheme (dendrogram) was developed, identifying two broad classes of families who opt for private education. Class 1: “families consolidated in the private sector” (62.63% of the sample) and Class 2: “families in transition to the private sector” (37.37% of the sample). This multiple correspondence analysis and hierarchical classification of families reveals the internal boundaries and differentiations within this subsector (Ziegler and Giovine, 2025).

## 3 Evolution of private education: privatisation of social mobility?

### 3.1 Historical evolution of enrolment and investment

**Figure 1:**
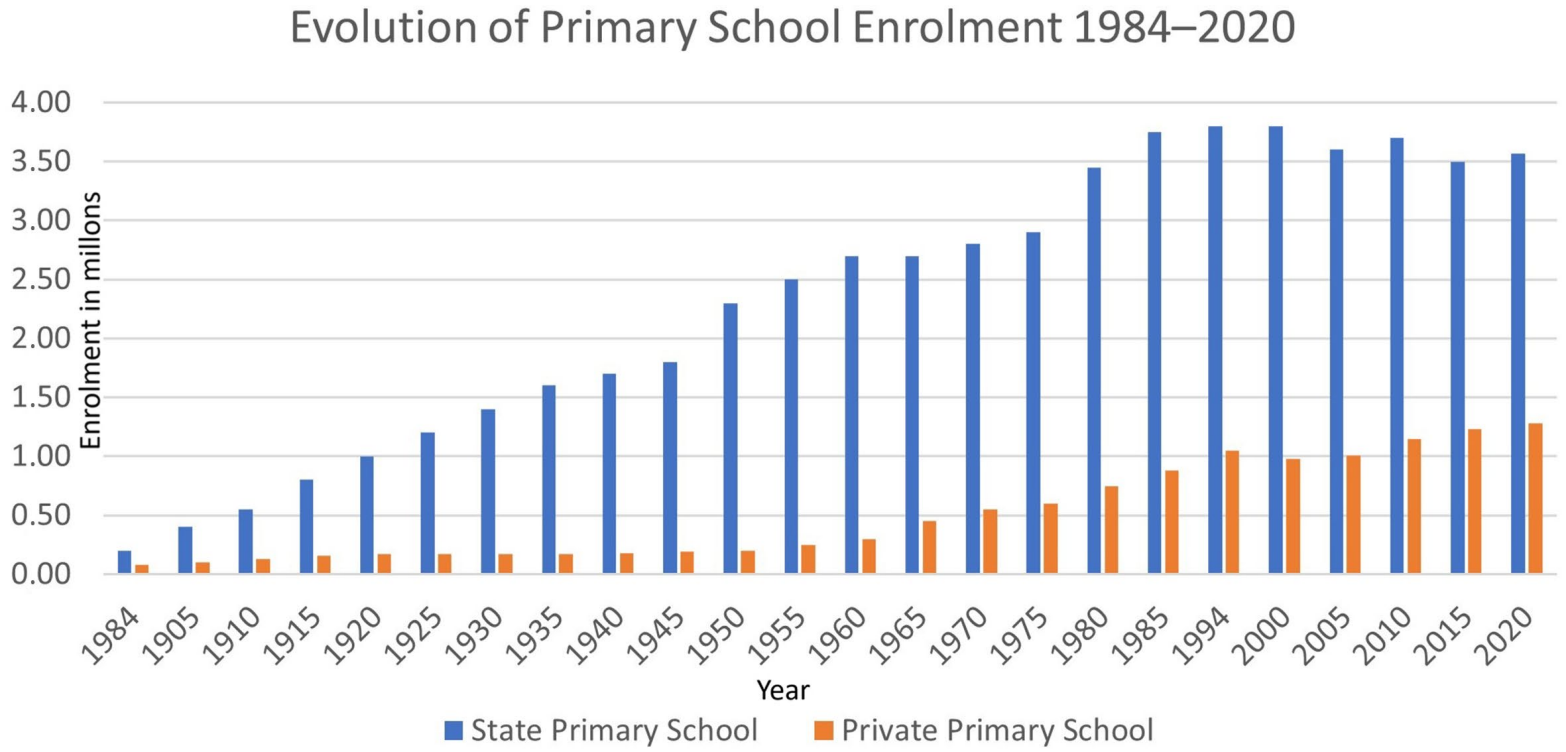
Evolution of primary school enrollment 1984–2020. Source: based on data from Gasparini et al. (2011), period 1984–2005, and Ministerio de Capital Humano (2024a)-Secretariat of Education, period 2005–2020.

Argentina is a country that has experienced significant growth in primary, secondary and higher education enrolment during the 20th century and so far in the 21st century. This growth has not occurred simultaneously at all levels. The primary level was characterised by an expansion in coverage by the state sector from the beginning and throughout the 20th century, reaching practically universal levels by the end of that period (see Figure 1). In this context, the private sector had a minority share until the mid-20th century, when it began to grow at a similar rate to the state sector, narrowing the gap between the sectors after the turn of the century.

**Figure 2:**
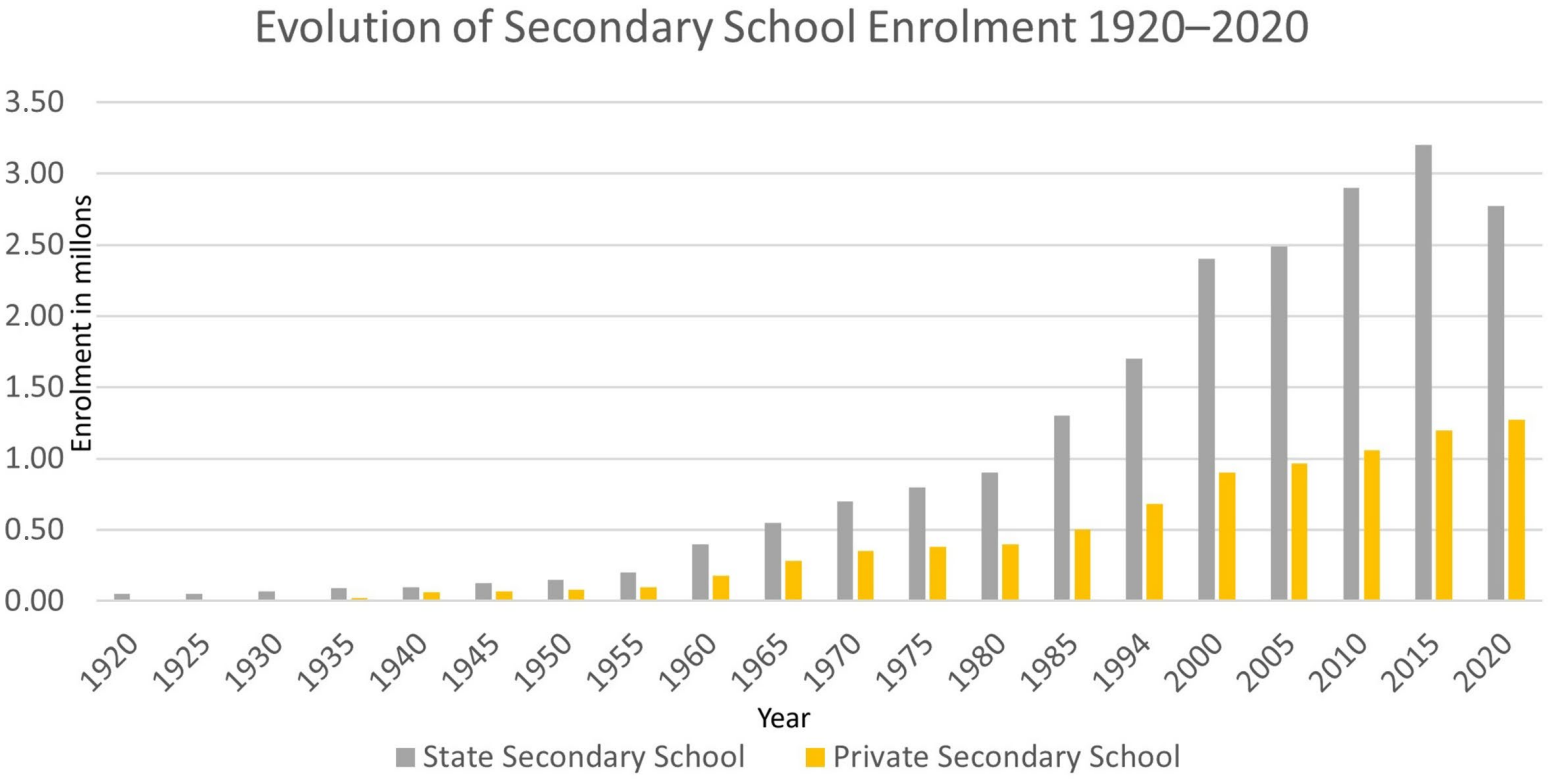
Evolution of secondary school enrollment 1920–2020. Source: based on data from Gasparini et al. (2011), period 1920–2005, and Ministerio de Capital Humano (2024b)-Secretariat of Education, period 2005–2020.

Secondary education, on the other hand, remained much more restrictive until the mid-20th century (see Figure 2), with a strongly class-based approach and a curriculum geared towards entry into higher education, mainly university. It was not until the mid-20th century that state and private enrolment increased (although state enrolment grew more rapidly) until the end of that century.

Understanding the structural conditions of enrolment and the process of massification of access to secondary education allows us to contextualise the processes that we will describe later.

**Figure 3:**
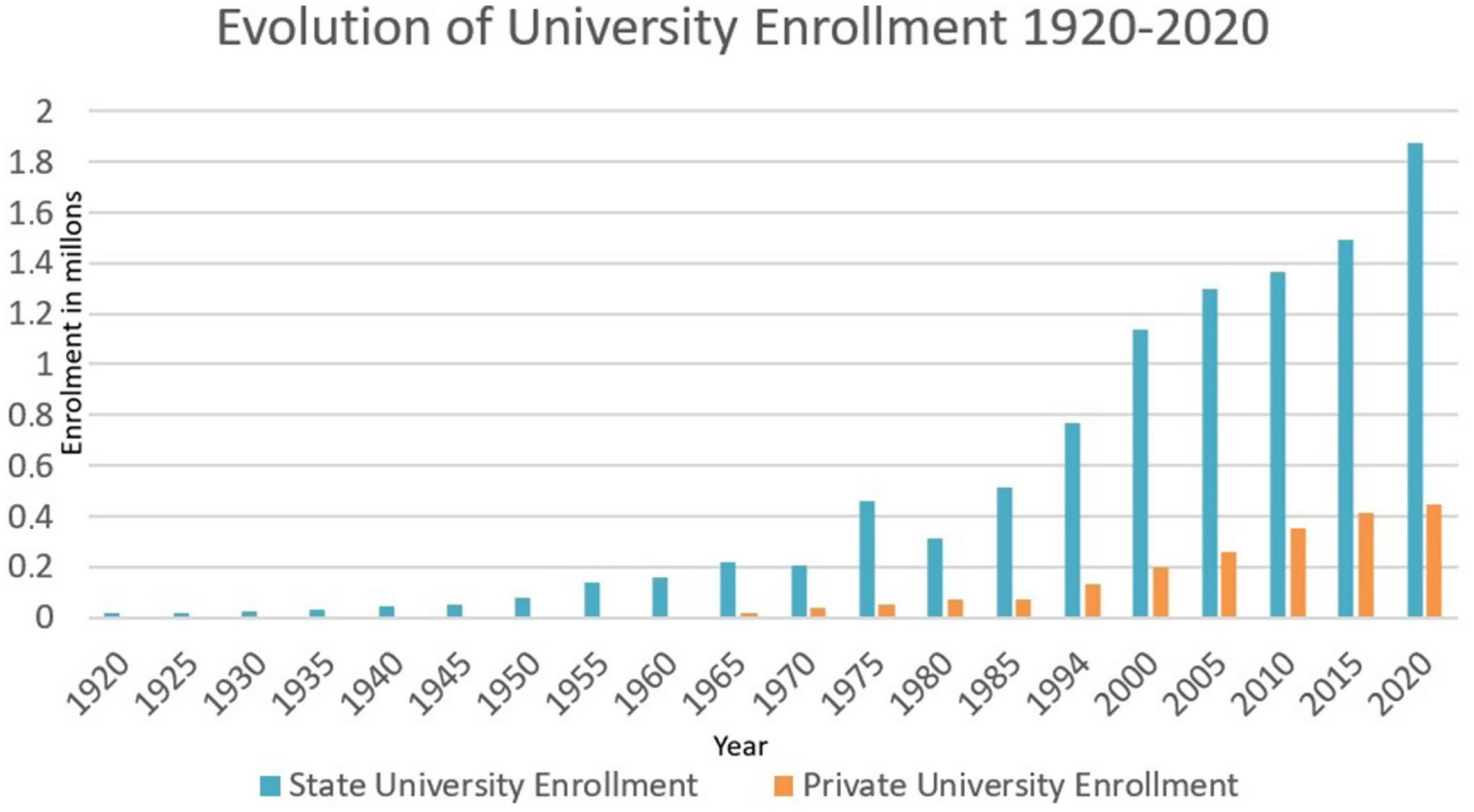
Evolution of university enrollment 1920–2020. Source: based on Cano (1985) period 1920–1983, Lodola et al. (2025) period 1983–2000, Ministerio de Capital Humano (2024c), 2000–2023.

University enrolment in Argentina follows a similar path to that of secondary education, with low figures until the mid-20th century, when growth began but stagnated in the 1950s with the military dictatorships, only to peak in 1975 and then fall sharply until the beginning of democracy (see Figure 3). At the same time, in the mid-20th century, private universities began to award professional degrees. With the arrival of democracy in the 1980s, enrolment grew in both sectors, with a higher proportion in the state sector.

One of the main indicators of the importance and priority of education in the national context is the financial resources invested in it. In an OECD report on inequalities in education investment among OECD member and partner countries, Argentina ranks extremely low, among the seven countries with the lowest investment, with expenditure per full-time equivalent student of less than $5,000 for primary to post-secondary non-tertiary education (OECD, 2025).

Comparing the income of full-time workers aged 25 to 64 with tertiary education in 2023 to those with upper secondary education in Argentina, the OECD results show that those with a short-cycle tertiary qualification obtain a relatively low return on their degree, around 25% more than those with secondary education. A bachelor’s degree yields a significant increase of approximately 75%, and a master’s or PhD degree increases income by 150%. This highlights the importance in terms of profitability that postgraduate education is having in the upper classes in Argentina as an instrument of distinction (Giovine, 2022).

It is important to note that, unfortunately, women’s incomes continue to be lower than men’s, even when controlled for educational level. However, this gap narrows as the level of education increases, reaching around 25% at the higher level, which makes education a proportionally more necessary and equalising resource for women (OECD, 2025).

### 3.2 School completion and performance: evidence of the hidden selectivity mechanism

Enrolment indicators for secondary education in Argentina show that around 70% of children of the theoretical age to enter secondary school actually attend, and more than 80% are already enrolled if we consider one year older than the theoretical age (OECD, 2025). Secondary education (which in Argentina runs from 12 to 17 years of age and includes lower and upper secondary education as compulsory) had an enrolment of more than 4.1 million students in 2024. This represents almost a third of enrolment in compulsory education (from 4 to 18 years of age—LEN 26.206). In this context, the net secondary school enrolment rate in Argentina is one of the highest in Latin America, at around 94% (Vázquez et al., 2025).

The most serious problem is observed in the completion of the level, which, although it has increased in the last 10 years (67% in 2014 and 74% in 2024), remains proportionally very low for lower-income sectors compared to higher-income sectors (60% in quintile 1 and 92.2% in quintile 5 in 2024, Postay et al., 2025). If we consider completion on time and in the proper manner, “only 10 out of every 100 students who started primary school in 2013 managed to reach the end of secondary school in 2024, that is, without repeating or dropping out and with satisfactory knowledge of language and mathematics” (Alzú et al., 2025: 01).

From first to fifth year, enrolment falls by 33% at secondary level. It falls by almost 40% when we consider only the state sector and only 15% in the private sector (Ministerio de Capital Humano, 2024a), so one might think that there is a lower percentage of dropouts and desertions in the private sector. In relation to this difference, it is worth mentioning that in 2017 a significant percentage of 16- and 17-year-olds in Argentina were engaged in productive activities, with 35.3% in urban areas and 56.8% in rural areas (Dirección Nacional de Evaluación, Información y Estadística Educativa, 2022).

Secondary school enrolment has grown by more than 10% in absolute terms in the 2010s, and this relative growth is greater in the private sector than in the state sector (Dirección Nacional de Evaluación, Información y Estadística Educativa, 2022). In 2024, the private sector accounts for 40% of total secondary enrolment, 42% of service units, 34% of sections and 30% of teaching positions (Ministerio de Capital Humano, 2024a).

These data indicate a lower percentage of students per service unit in the private sector, but a higher percentage per average section in the country. On the other hand, private service units in 2019 had a higher proportion of internet access, availability of connection in classrooms, days of classes actually taught and, in 2018, a lower proportion of students who drop out and do not advance to the next school year because they do not meet the minimum academic requirements. Furthermore, indicators show that teachers in the private sector have less teacher training and more undergraduate and postgraduate university education (Dirección Nacional de Evaluación, Información y Estadística Educativa, 2022).

### 3.3 Segmentation and increase in private demand

Argentina has a highly segmented school system in terms of enrolment composition (segregation by management sector, state, subsidised private and fully private) and fragmented in symbolic terms, in terms of the formation of ghettos at the social extremes where socialisation is closed among students with very similar characteristics (Giovine and Arce Castello, 2024). The attendance of families of disadvantaged students in the state sector is 35% and in the private sector practically 8% in 2022, which places the country at intermediate levels in the Latin American context [Murillo et al. (2025), which is already a highly segregated region worldwide].

**Figure 4:**
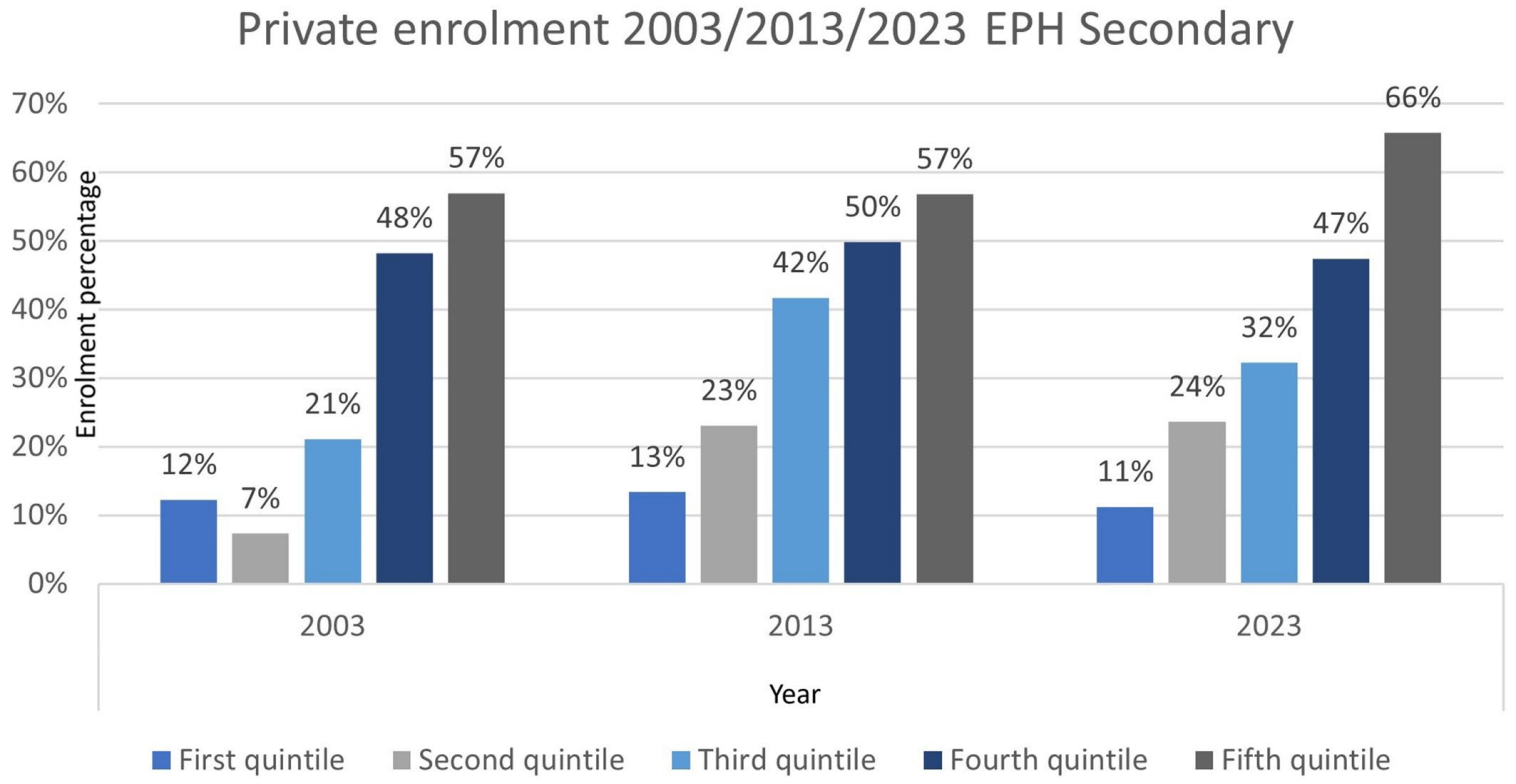
Private enrollment 2003/2013/2023 EPH. Source: based on data from the Permanent Household Survey—INDEC 2003, 2013, 2023.

Taking data from the Permanent Household Survey (EPH), which allows us to analyse the demand for private education in large urban agglomerations, in Argentina since the beginning of the 21st century we can see (see Figure 4) that the first quintile (quintiles are considered based on *per capita* family income) has maintained its share in the sector at around 12%, while the second quintile has practically tripled its share, going from 7% in 2003 to 24% in 2023. This sector is one of the main recipients of lower resources from the private secondary education system. On the other hand, the third quintile also doubled its share from 2003 to 2013, but then fell back to 32%. The fourth quintile remained relatively stable at 50% of cases, but the highest income quintile increased by 10% from 2013 to 2023.

### 3.4 The compensatory effect of private school attendance on learning

The information to be analysed is based on data from the Operativo Aprender (Learning Assessment) conducted by the Ministry of Education. The results of the latest assessment in 2024 show that student performance in language area in state and private education is significantly different, with more than twice as many students below the basic level in the state sector (19.2% versus 8.7%). If we analyse the other extreme, students who obtain an advanced percentage, we see that the private sector practically triples the state sector (11.5% versus 3.9%). In mathematics, the situation is more serious; with the same trend, the absolute values are even higher. In the state sector, 62.2% do not reach the basic level, while in the private sector the percentage is much lower (38.7% do not reach it). In contrast, when we look at the satisfactory level in mathematics, 24.2% achieve it in the private sector, while only 9.4% do so in the state sector. In both cases, the percentages at the advanced level are very low, but in the state sector they are practically three times lower (Ministerio de Capital Humano, 2025).

**Figure 5:**
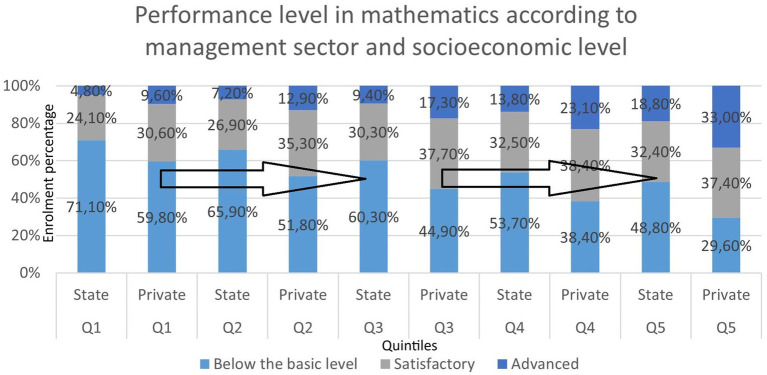
Performance level in mathematics according to management sector and socioeconomic level secondary. Source: Ministerio de Capital Humano (2025): 33, based on data from the 2024 Aprender assessment, DNEIEE-REFCEE.

These indicators are even more worrying when performance is looked at by socioeconomic level (see Figure 5). In language, the poorest quintile (here, the quintiles are constructed using a multidimensional methodology that reflects characteristics of the economic level and educational climate of the household, Ministerio de Capital Humano, 2025: 86) scores 22.9% below basic, while only 1.9% are at advanced level. In the richest quintile, the percentage below the basic level is 10.2%, while 12.7% achieve an advanced level, almost 10 times more than the poorest quintile. In mathematics, the situation is even more acute, as might be expected: 70% of students in the first quintile perform below the basic level, while 36.3% of the richest quintile, practically half. In contrast, 28% of the latter quintile perform satisfactorily, compared to 5.2% of the poorest quintile, about five times less (Ministerio de Capital Humano, 2025).

**Figure 6:**
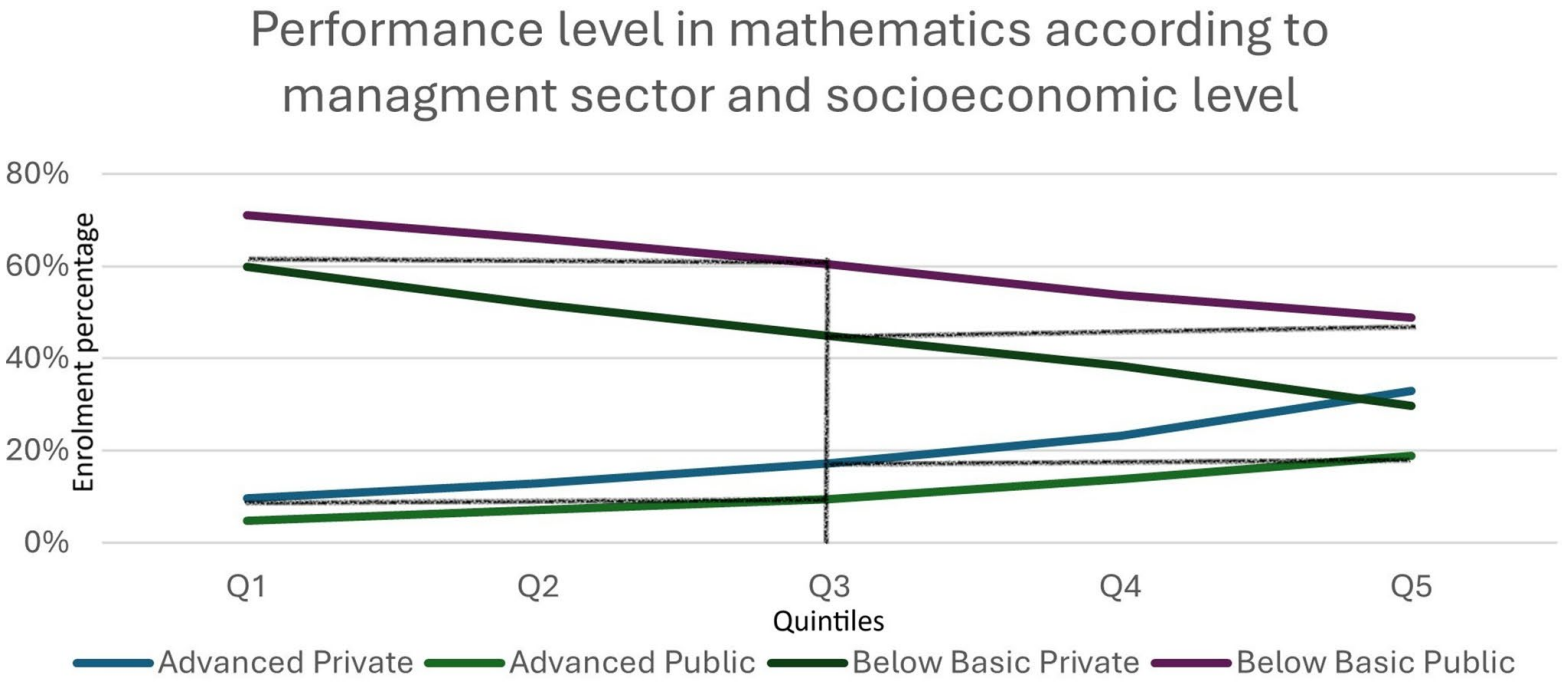
Performance level in mathematics according to management sector and socioeconomic level. Source: Ministerio de Capital Humano (2025): 33, based on data from the Aprender 2024 assessment, DNEIEE-REFCEE.

Figure 6 shows that, among students in the poorest quintile, attendance at private schools is associated with an increase of approximately two deciles in mathematics scores.

In relation to this last piece of data, the comparison of mathematics performance by quintile and by management sector allows us to isolate the effect of economic capital. Regardless of economic level, the private sector continues to perform better. The poorest quintile increases performance below the basic level in the state sector by 11.3%, while in satisfactory performance it doubles, rising from 4.8 to 9.6%. This is a clear indicator that the private management sector is generating bett sults in the most impoverished groups. This difference increases from quintile to quintile up to the highest income quintile, where the private sector achieves a satisfactory performance of 33%, compared to 18.8% for the state sector.

The results show that access to the private management sector guarantees better scores in both language and mathematics. It could be argued that this is because the children of families with higher incomes attend private schools, but when controlling for income quintiles, these differences remain. Moreover, there is a tendency for the difference between the private and state sectors to increase as income per quintile increases.

This increase in the difference would account for better comparative performance in terms of learning as income per quintile increases in the private sector. We can put it this way: if we compare the mathematics performance of the poorest (first quintile) who attend private schools, it is practically equivalent to the mathematics performance of third quintile students who attend state schools (see Figures 5, 6). This means that, considering only these isolated indicators and knowing that a systemic view would be better, first quintile families who send their children to private schools improve their performance by two quintiles compared to families who send their children to state schools.

This comparison can also be made with third quintile families who send their children to private schools, who achieve similar results to fifth quintile families who send their children to state schools. Again, the differences in mathematics performance are approximately two quintiles per management sector. We have chosen mathematics performance because it is the most widely referenced internationally (León and Youn, 2016; Paredes Mamani, 2015; Zambrano, 2016, among others), but if we consider language performance controlled by income quintiles, the results are similar.

## 4 The social space of demand for private education: internal cleavages

### 4.1 Differentiation among families: established versus transitioning to private education

As has been shown, enrolment in the private sector is a heterogeneous subsector. Based on surveys and multiple correspondence analysis (MCA), a hierarchical classification of families who choose private education has been established, revealing two main classes: Class 1: “families consolidated in the private sector” (62.63% of the sample) and Class 2: “families in transition to the private sector” (37.37% of the sample) (Ziegler and Giovine, 2025).

*Consolidated families*, with at least two previous generations in private institutions, have higher institutionalised cultural capital, evidenced by the high levels of parental qualifications (university or postgraduate). Within this group, two subclasses can be distinguished: one that seeks a globalised and exclusive education for their children, predominantly with a high investment in secular schools and without a subsidy. Subsidies for private education in Argentina consist of financial support provided by the state to private schools (which may be total or partial and is mainly intended for religious schools, Catholic schools and others). The other subclass prioritises vocational training in partially subsidised institutions, with a more pragmatic approach and lower financial investment. These families, who are well established in private education, pursue a strategy of greater social closure (Parkin, 2001) due to the selective nature of the institutions they attend.

*Families in transition*, on the other hand, represent the first generation to access private education, which in some cases only begins at the secondary level for their children. These families have lower levels of education among their parents (secondary or primary education) and tend to opt for private religious schools, with high percentages of state subsidies. Proximity to home and the search for an education in religious values are important factors in their choice. Two fractions can be distinguished within this group: one with better technological and housing resources, which prioritises education in religious values, and another with fewer resources, which mainly seeks the status associated with private schools, as these institutions are located in the neighbourhoods where they live and stand out from the state-run options available. In the first group, families have higher levels of education than in the second (especially mothers who may have completed non-university higher education, so these are families with mothers who are teachers). A study for Brazil by Perosa and Dantas (2017) yields a relatively similar result in that it suggests that formal employment of mothers is an important predictor of the choice of private school. In this sense, in our research, the completion of non-university higher education and teaching work is a condition that is observed in the choice of private sector institutions among families in this analysed group.

The analysis of the identified groups allows us to investigate the dynamics of the transition to private education, given that in all cases there is a previous generational trajectory in the state system. As we have mentioned, in groups with higher levels of qualifications who choose schools with higher fees, there is usually a longer intergenerational presence in the private sector. In contrast, in families with more recent entry into private education, we find that one of the parents (not both) has a background in this subsector. In cases of more recent entry, where there are greater economic constraints, state education has a more marked presence among parents and their children, both in the imagination and in the immediate educational experience they mention.

### 4.2 Characterisation of “families on the threshold of private education”

Within class 2, the most relevant subclass for analysing the mobility of the lowest quintiles is the one called “families on the threshold of private education” (Ziegler and Giovine, 2025), which represents 8.7% of the families surveyed. Their material conditions reflect the economic constraints they face, despite their choice of private school: they live in homes with few rooms for exclusive use and have limited access to computer resources, as they do not have computers and connect predominantly via mobile phones.

This group is characterised by having the lowest cultural and economic capital. In some cases, their parents have only completed primary education, and mothers rarely complete secondary education. The transition to private education for this subclass is a strategy for change in the family’s educational trajectory, as both responsible adults have studied in state schools.

Despite these limitations, these families attend private institutions that are fully subsidised by the state. These schools therefore have more affordable fees than the average private school and are located in large cities. Although they pay a symbolic amount, the financial effort they make to send their children to a private school is very significant. Education expenditure can represent a very significant outlay in their budget, with between 31 and 50% of their monthly income going towards school fees. This data highlights the sacrificial nature of the choice, which seeks to maximise educational opportunities at the cost of a significant budgetary effort.

### 4.3 Motivations and strategic duality in the face of selectivity

The motivations for choosing the private sector among families on the threshold of private education are strongly linked to the search for continuity (no interruption of school days due to teacher strikes), predictability and prestige in a system perceived as unstable.

This subclass chooses the private schools their children attend because they confer status. In addition, they seek academic guarantees; they agree that private schools offer an institutional project that is sustainable over time, higher quality and academic rigour. The search for this guarantee of quality explains the correlation between the choice of private education and the two-quintile increase in academic performance observed in our quantitative data presented in the previous sections.

It is crucial to note that, for these groups with less capital, the provision of religious education acts as a “gateway” to the private sector, as these institutions generally receive higher state subsidies and their fees are affordable. However, affiliation with religious education is not always part of their core values; in some cases, these schools are simply the most accessible, serving as a gateway to private education after recently leaving state schools.

Perceptions of state schools reveal a tension in the choice. Due to their own background, “families on the threshold” maintain close ties with state education. One of the virtues they value is that “they do not treat you like a customer.” However, they see a flaw in its secular nature and do not agree that state schools provide a better academic standard. This reinforces the idea that the choice is a pragmatic calculation: they use the private system to obtain the knowledge to continue their higher education at university.

A paradigmatic case to illustrate this group is a family of more than six members with significant material constraints (both in terms of housing and access to technological resources) that characterise this high-effort strategy. They indicate that they choose private school (fully subsidised) because of the values offered by the institution, the future prospects of its graduates, and its geographical location. This family has high expectations of the school, believing that it provides “openness to the world, job skills, knowledge of a foreign language (an aspect that is practically absent from state education), continuation in higher education, general culture, entry into the labour market, a network of contacts, good citizenship, status and institutional belonging”.

This family’s home has low institutionalised cultural capital. With the exception of the mother, who has completed tertiary education, the father has completed primary education; the grandparents also only reached primary level. Both adults attended state schools, so this is a recent transition to the private sector, which began at secondary level for their son, after he attended primary school at a state school. There are family networks of siblings and cousins at the chosen school, but no history of previous generations attending.

The choice of school was a collective process within the household, based on comments from acquaintances and information obtained through social networks. They evaluated both state and private options. The main reasons for their choice were institutional values, the future prospects of graduates and the geographical location. For the family, private school guarantees continuity of classes, institutional predictability, extended hours, better resources and infrastructure, committed families and a more homogeneous social environment.

This family, which invests a high percentage of its income in subsidised private secondary education to achieve better performance, maintains that it wants its children to continue their studies at a state university. This strategic duality (the private sector to obtain selective quality in secondary education, combined with the state sector for free tuition and prestige at university) illustrates how these groups develop options in the face of the veiled selectivity of the education system. Attending private secondary school encapsulates the strategy of obtaining an academic preparation that will enable the transition to higher education, which, although it offers open and free admission, produces a selection in the early years based on academic performance.

## 5 Final discussion

The analysis presented in this article allows us to understand how Argentine social structure is traversed by a persistent tension between a deeply rooted egalitarian imaginary and the economic, political, and social transformations that have eroded its material foundations, reconfiguring the place of education in the processes of social mobility. The social matrix that consolidated throughout the 20th century gave rise to the image of an expanding middle class society, where increasing educational credentials were seen as a privileged route to social advancement. However, the changes that began in the 1970s and deepened during the 1990s altered this equation, giving rise to phenomena such as “spurious mobility” (Kessler and Espinoza, 2003), in which occupational advancement does not necessarily translate into improvements in income or job stability. Education, although it retains a strong symbolic value, no longer guarantees quality jobs or sustained well-being on its own.

In this context, the Argentine education system is shaped by a central paradox. On the one hand, it maintains an open access model that allows free enrolment at all levels (including university) without selective entrance exams and under a principle of free education that retains strong social legitimacy. On the other hand, its actual functioning is organised through veiled selection mechanisms that operate throughout school careers, mainly in the form of dropouts, repetition and the inability to meet academic performance requirements, which shows the extent of this “soft” selectivity (Di Piero, 2022). Inequality by income level is particularly striking: while completion rates reach 60% in the first quintile, they rise to 92.2% in the highest income quintile.

In this scenario, the choice of private education at secondary level emerges as a pragmatic response to the fear of social decline (Ziegler and Giovine, 2025). Far from being limited to the highest income groups, this strategy extends to the middle and lower sectors. Data from the Permanent Household Survey show that the second income quintile has nearly tripled its share of private enrolment in the last two decades, rising from 7 to 24%. The shift towards the private sector does not represent a break with the egalitarian ideal, but rather a way of sustaining it. In a formally open system, families seek to “ensure” continuous and successful school trajectories that enable access to higher levels, even at the cost of significant financial effort.

An analysis of academic performance helps explain the rationale behind these decisions. When controlling for mathematics learning by income quintile, the privately managed sector consistently achieves better results, even among the most impoverished groups. Students in the first quintile who attend private schools achieve performance levels comparable to those in the third quintile who attend state schools. The improvement equivalent to two quintiles of performance reinforces the rationale for this choice, particularly for “families on the threshold of private education” (Ziegler and Giovine, 2025), who may spend between 31 and 50% of their income on school fees. The economic sacrifice is justified in the name of academic gain, which is perceived as a prerequisite for sustaining expectations of upward mobility.

However, this individual quest for protection against the risk of downward mobility has structural effects. The expansion of private enrolment (largely subsidised by the state) accentuates the segmentation of the education system. Family strategies that seek to anchor social reproduction through better educational opportunities encourage processes of social closure and contribute to the formation of relatively homogeneous circuits, both in the state and private sectors. The evidence presented shows that the Argentine school system is highly segmented in terms of social composition and strongly fragmented on a symbolic level, giving rise to increasingly differentiated school experiences and socialisation among peers with similar characteristics.

From this perspective, private education cannot be approached as a homogeneous block. Analysis of the social space of demand reveals the existence of internal cleavages: on the one hand, families consolidated in the private sector, with high levels of cultural and economic capital, who guide their children towards more selective and expensive institutions, reinforcing strategies of social closure; on the other hand, families in transition and, within them, “families on the threshold of private education,” who access subsidised religious schools with relatively low fees but which involve a huge budgetary effort. In these cases, the transition to the private sector constitutes a break with previous trajectories in state schools and encapsulates a commitment to educational continuity and access to state universities as a horizon for future improvement.

Overall, the results of the study show that open access to the education system coexists with covert selection mechanisms that are linked to class structure and the different strategies of families. Private secondary education functions simultaneously as a buffer for covert selectivity (by improving the performance of lower-income sectors that manage to enter this circuit) and as a device for reproducing inequalities, by contributing to school segregation and reinforcing existing stratification. In this sense, the meritocratic ideal that organises much of the social representations of mobility is strained: school “meritocracy” is based on increasingly unequal schooling opportunities.

The tensions and paradoxes identified open up several lines of research. First, it is essential to develop longitudinal studies that follow the trajectories of families on the threshold of private education, to investigate whether the improvement of two quintiles in performance effectively translates into access, permanence and graduation in higher education, and to what extent this is linked to processes of social mobility and sustain e upward mobility over time. Secondly, it is essential to analyse in greater depth the effects of state subsidies to the private sector on segregation: to what extent does this funding contribute to reducing gaps or, on the contrary, consolidate educational segmentation? Finally, it is necessary to advance research on symbolic segregation and the social capital built in these differentiated circuits, examining how the social closure of “established families” and the acceptance of homogeneous environments by “families on the threshold of private education” shape students’ networks of contacts, future opportunities, and horizons of possibility.

Going beyond merely noting educational inequalities, this work proposes an interpretation that links the veiled selectivity of the educational system with family strategies and the place assumed by the private sector in contemporary Argentina. In a society historically defined by its promise of upward mobility, understanding these mechanisms is essential for devising policies that address not only access, but also the specific conditions of schooling and the selection mechanisms that silently update and reconfigure the ways in which inequality is reproduced.

## Data Availability

The original contributions presented in the study are included in the article/supplementary material, further inquiries can be directed to the corresponding author.
